# Supplementary material for: Reliability of the use of foot pressure pain threshold in adults: a test-retest analysis
Source: PeerJ. 2025 Aug 12;13:e19875. doi: 10.7717/peerj.19875 (PMC12356193; doi:10.7717/peerj.19875)
Supplement: Supplementary file 1 [file peerj-13-19875-s002.odt]

CHECKLIST

TITLE AND ABSTRACT

1. Identify in title or abstract that interrater/intrarater reliability or agreement was investigated.

Reliability of the use of Foot Pressure Pain Threshold in adults: A test-retest analysis.

INTRODUCTION

2. Name and describe the diagnostic or measurement device of interest explicitly.

Pressure pain thresholds (PPT)

Tool: JTECH Commander digital algometer.

3. Specify the subject population of interest.

Adults: aged 55 to 75

4. Specify the rater population of interest (if applicable).

Volunteers regularly attending a podiatry clinic were invited to participate in the study.

Inclusion criteria aged 55 to 75 and exclusion criteria amputation of one or both lower limbs, ulcers or open wounds on the soles of the feet,calcaneal spurs, plantar fasciitis (acute foot pain), or were undergoing systemic corticosteroid treatment.

5. Describe what is already known about reliability and agreement and provide a rationale for the study (if applicable).

The digital pressure algometer is a diagnostic tool used to assess pain thresholds in patients, especially in musculoskeletal conditions like fibromyalgia, myofascial pain, and joint discomfort.  It is noted that the digital algometer has several advantages, such as being easy to use and providing accurate and reliable measurements (Jones et al., 2007; Nussbaum E.L. & Downes L., 1997). The  algometer displays the pressure pain threshold (PPT), which is the minimum pressure applied to  the evaluated area that triggers pain (Fischer, 1987; Kinser et al., 2009; Saban & Masharawi, 2016). It is important to note that the reliability of PPT data depends on the technique used by the observer and the individual's ability to provide clear verbal feedback on their perception of PPT levels (Chesterton et al., 2007). Moreover, pain measurement can be useful for tracking its progression  over time or as a predictive tool for future outcomes (Walton et al., 2011).

METHODS

6. Explain how the sample size was chosen. State the determined number of raters, subjects/objects, and replicate observations.

A minimum of 25 participants were required to ensure reliable calculations, based on an alpha level of 0.05, power (P) of 0.8, and an expected ICC1,1 greater than 0.85 (with a lower bound of the 95% confidence interval at 0.65) (Mondal et al., 2024). Given that the study involved two appointments spaced one week apart, and there was a potential risk of participant drop-out between sessions, 32 volunteers were initially recruited to secure the desired sample size. On the day of the first session, 31 volunteers attended.

1 evaluator.

Each measurement at each point was taken three times, with the same procedure repeated after 30 minutes and again after seven days.

7. Describe the sampling method.

The pressure algometer was employed to objectively assess the foot pain threshold, defined as the minimum tolerable pressure before pain is perceived. The specific algometer used for these measurements was the Algometer Commander from JTECH Medical, manufactured  in 2004. This instrument featured a 1 cm probe for measurement, using Newtons (N) as the unit of  measurement (Saban & Masharawi, 2016). During the procedure, the patient was positioned comfortably in a supine position on an examination table. Measurements were taken on both feet, the right (R) and the left (L), at four specific points: the first metatarsal head (1st MH), fifth metatarsal head (5th MH), first toe pulp  (1st TP), and heel). For each point, the probe was placed perpendicularly, and pressure was applied gently to the area.

8. Describe the measurement/rating process (e.g. time interval between repeated measurements, availability of clinical information, blinding).

Each measurement at each point was taken three times, with the same procedure repeated after 30 minutes and again after seven days.

9. State whether measurements/ratings were conduced independently.

3 measurements were taken independently for each participant.

10. Describe the statistical analysis.

Descriptive analyses were used to report the characteristics of the participants. The assumption of  normality was inspected visually and tested using the Shapiro-Wilk test. Normally distributed data  were presented as mean (SD). All continuous data were analyzed using an independent t-test (for normally distributed data) or the non-parametric Mann-Whitney U test. The Chi-squared test was  used for categorical data.

RESULTS

11. State the actual number of raters and subjects/objects which were included and the number of replicate observations which were conducted.

1 evaluator

31 participants

3 sessions

12. Describe the sanple characteristics of raters and subjectis (e.g. training, experience)

an experienced podiatrist.

13. Report estimates of reliability and afreement including measures of statistical uncertainty.

Reliability ranged from good to excellent (ICC: 0.76 to 0.91)

DISCUSSION

14. Discuss the practical relevance of results.

The digital algometer is an essential tool for measuring pain thresholds. Its accuracy, validity, reliability, and ease of use make it an excellent choice for assessing pain thresholds, enabling more effective diagnosis and follow-up for patients with pain-related conditions. Moreover, its application has the potential to enhance clinical interventions and provide objective data that can inform the development of new therapeutic strategies.

AUXILIARY MATERIAL

15. Provide detailed results if possible (e.g. online)

Article with database in supplementary material
